# Cholecalciferol supplementation lowers leptin and TMAO but increases NO and VEGF-A levels in obese vitamin D deficient patients: Is it one of the potential cardioprotective mechanisms of vitamin D?

**DOI:** 10.1186/s12986-022-00666-4

**Published:** 2022-04-29

**Authors:** Mateusz Ozorowski, Michał Wiciński, Łukasz Wróbel, Anna Fajkiel-Madajczyk

**Affiliations:** Department of Pharmacology and Therapeutics, Faculty of Medicine, Collegium Medicum in Bydgoszcz, Nicolaus Copernicus University, M. Curie 9, 85-090 Bydgoszcz, Poland

**Keywords:** Vitamin D, Inflammation, Obesity, Cardiovascular diseases, Markers, NO, Leptin, TMAO, VEGF-A, sST2

## Abstract

**Background:**

Vitamin D deficiency is one of the most common health issues in developed countries. Obese patients are most at risk of having serum 25-hydroxyvitamin D_3_ (25(OH)D_3_) levels that are too low due to the accumulation of vitamin D in adipose tissue. While the effects of a deficiency on the skeletal or immune system are known, the effects on the cardiovascular system are not yet clear. Our study investigates the effect of cholecalciferol supplementation in obese patients on selected biomarkers associated with cardiovascular diseases (CVDs).

**Methods:**

The study enrolled 33 obese patients with insufficient 25(OH)D_3_ levels. For three months, the subjects supplemented with cholecalciferol at a dose of 2000 IU/day. Concentrations of nitric oxide (NO), vascular endothelial growth factor A (VEGF-A), leptin, trimethylamine N-oxide (TMAO) and soluble suppression of tumorigenicity 2 (sST2) were measured in baseline samples using ELISA (BioTek EPOCH). 25(OH)D_3_ levels measured on Beckman Coulter DXI 800 by chemiluminescence method.

**Results:**

After supplementation, 25(OH)D_3_ levels increased significantly. Normal levels were achieved in most patients. A statistically significant reduction leptin and TMAO levels was observed. At the same time, NO and VEGF-A levels increased statistically significantly.

**Conclusion:**

This study indicates that restoring normal 25(OH)D_3_ levels in obese people reduces the concentration of pro-inflammatory factors associated with cardiovascular diseases. Reducing inflammation and the potential impact on vascular reactivity leads to the conclusion that cholecalciferol supplementation in obese patients may benefit the cardiovascular system.

## Introduction

The vitamin D receptor (VDR) is located in endothelial cells, vascular smooth muscle and cardiomyocytes [1]. Many studies have described the importance of vitamin D deficiency in the development of atherosclerosis, coronary heart disease, hypertension, heart failure and atrial fibrillation. Moreover, the cardioprotective role of this vitamin in patients after myocardial infarction has been described [2].

There is evidence that vitamin D can modulate the pathogenesis of atherosclerosis. An undoubted role in the development of atherosclerotic plaque is played by a chronic inflammatory process. Inflammation together with oxidative stress promotes impaired vascular perfusion, resulting in an increased risk of coronary artery disease [3]. Tare et al. observed that mesenteric arteries of 25(OH)D_3_ deficient rats were characterised by a twofold decrease in their ability to diastole. The pathomechanism of this process is related to impaired NO signalling and endothelium-derived hyperpolarizing factor (EDHF) [4, 5]. In the *Framingham Offspring Study*, which included 1739 subjects without heart disease, the risk of a cardiovascular incident was 53% to 80% higher in subjects with low 25(OH)D_3_ levels during a seven-year prospective observation [6]. Anderson et al. reported that American patients with vitamin D deficiency, defined as serum 25(OH)D_3_ levels < 30 ng/ml, reached 60% and significantly correlated with the occurrence of type 2 diabetes, hypertension, coronary heart disease (CHD), myocardial infarction (MI), heart failure (HF) and was associated with higher overall mortality [7].

Vitamin D deficiency likely leads to the development of cardiovascular diseases (CVDs) by an overactive renin–angiotensin–aldosterone system (RAAS). It has also been proven that low serum 25(OH)D_3_ concentration is associated with endothelial dysfunction and increases inflammation [8].

Many studies have shown that obese people have lower levels of 25(OH)D_3_ the serum compared to people of normal weight. A potential explanation for this phenomenon is vitamin D sequestration in adipose tissue [9]. Another hypothesis is that decreased 25(OH)D_3_ levels in obese people are due to a sedentary lifestyle and lack of physical activity. It is associated with lower exposure to sunlight and reduced skin synthesis [10]. Targher et al. suggest that lower 25(OH)D_3_ levels may be associated with impaired 25-hydroxylation in non-alcoholic fatty liver disease (NAFLD), which is common in obese patients [11].

We can distinguish two types of adipose tissue—white adipose tissue (WAT) and brown adipose tissue (BAT) [12]. Vascular endothelial growth factor A (VEGF-A) is indicated as an important protein in the development of BAT, which shows increased metabolism in contrast to WAT [13]. WAT performs an auto, para- and endocrine function, and the substances it secretes, called adipokines, are pro-inflammatory and anti-inflammatory. The pro-inflammatory adipokines include leptin, tumor necrosis factor (TNF-α), resistin, interleukin 6 (IL-6) and visfatin [14]. Obesity is associated with chronic inflammation that results from excess body fat [15, 16]. Recent clinical studies show a positive correlation between increased serum levels of trimethylamine N-oxide (TMAO) and an increased risk of adverse cardiovascular events. There is compelling evidence suggesting a relationship between TMAO and inflammation [17]. Studies have suggested that not only low vitamin D level but also high level of TMAO, which is associated with changes in the gut microbiota of obese individuals, are associated with the severity of NAFLD [18].

Nitric oxide (NO) plays an important role in regulating blood flow and pressure [19]. It is produced not only in the endothelium, but also in cardiomyocytes, smooth muscle cells, monocytes and macrophages, and in thrombocytes. It has an anticoagulant and antiplatelet effect by inhibiting the formation of the active GPIIb/IIIa receptor conformation, and affects the contractility of cardiomyocytes [20].

Leptin is a protein hormone belonging to the group of adipokines, produced by adipocytes. This peptide has been shown to activate the renin–angiotensin–aldosterone axis (RAA), increase the reabsorption of sodium in the renal tubules and stimulate the activity of the sympathetic nervous system [21].

The relationship between VEGF-A and the heart is two-sided. On the one hand, VEGF-A activates cardiomyocytes, inducing morphogenesis, contractility and wound healing. The concentration of VEGF-A increases in cardiomyocytes during inflammation and mechanical damage to the heart. Moreover, high concentrations of VEGF-A have been found in patients suffering from various CVDs, which are often correlated with poor prognosis and disease severity [22].

A new biomarker of heart failure is the ST2 (suppression of tumorigenicity 2) receptor, which belongs to the interleukin 1 (IL-1) receptor family. Of the 4 known isoforms of this glycoprotein, 2 play a special role in the physiology and pathophysiology of the cardiovascular system: transmembrane (ST2L) and soluble (sST2), present in the blood [23]. A meta-analysis by Ip et al. showed that sST2 significantly predicts severity and mortality in cardiovascular diseases and is a good predictor of mortality in patients with stable coronary disease and chronic heart failure [24].

The results of studies available in the literature on the discussed biomarkers remain inconclusive. We decided to investigate the effect of cholecalciferol supplementation in obese patients on the concentration of biomarkers with their potential role in CVD.

## Material and methods

The study population consisted of selected patients of Primary Care Clinic in Poland. Inclusion criteria for the study were: age (subjects over 18 years old) and obesity (according to BMI). Exclusion criteria for participation in the study were: nicotinism, hormone replacement therapy, history of myocardial infarction or stroke within the past year, cancer, dialysis, liver disease, osteoporosis, pregnancy, vitamin D malabsorption (cystic fibrosis, Crohn's disease), allergy to components contained in the tablet of the drug, refusal to draw blood for the study. All subjects were in good health and were not on any special diet. The study was conducted between October 2019 and March 2020, eliminating the effect of UV-B radiation on dermal cholecalciferol synthesis. The study design was approved by the ethics committee of Collegium Medicum in Bydgoszcz, Nicolaus Copernicus University, in Toruń (approval number KB48/2019). The study was conducted according to the criteria set by the declaration of Helsinki and each subject signed an informed consent before participating in the study.

42 patients were included in the study and consented to participate in it. Nine dropped out during the study or did not show up for repeat blood draws. 33 patients (17 males and 16 females aged, 23–71) completed the study. Table 1 shows basic anthropometric data of the subjects.

**Table 1:** Anthropometric data of patients

	Baseline	After 3 months of supplementation
*Male*		
Number	17	
Age (mean)	40.59	
BMI (kg/m^2^)	37.85 ± 5.92	37.98 ± 6.59 (*p* = 0.651)
*Female*		
Number	16	
Age (mean)	47.69	
BMI (kg/m^2^)	35.92 ± 6.10	35.6 ± 6.33 (*p* = 0.052)

Patients were screened for serum vitamin D level, determined as 25(OH)D_3_, and for markers such as NO, VEGF-A, leptin, TMAO and sST2. The criterion for serum 25(OH)D_3_ deficiency was a serum concentration < 30 ng/ml. Subsequently, patients received cholecalciferol at a dose of 1000 IU (25 µg) per tablet. 180 tablets were the amount needed for 90 days of treatment. They were advised to take two tablets once daily after a meal in the morning for 3 months. After this time, serum 25(OH)D_3_ and marker levels were controlled again (Table 2).

**Table 2:** Statistical data before and after supplementation

	Mean ± SEM [1]	Mean ± SEM [2]	p value	Δ
NO (µmol/l)	39.19 ± 10.96	70.02 ± 13.80	0.021	+ 30.83 ± 12.38
Leptin (ng/ml)	16.90 ± 1.65	14.72 ± 1.78	0.029	− 2.18 ± 0.95
TMAO (ng/ml)	63.41 ± 12.59	59.98 ± 12.36	0.022	− 3.44 ± 1.42
VEGF-A (pg/ml)	298.81 ± 27.44	322.91 ± 26.02	0.024	+ 24.10 ± 10.13
sST2 (ng/ml)	46.75 ± 4.91	51.69 ± 4.28	0.065	+ 4.94 ± 2.59
25(OH)D_3_ (ng/ml)	18.22 ± 1.10	29.89 ± 1.16	< 0.001	+ 11.67 ± 1.05

Body mass index (BMI) was calculated as weight in kilograms divided by height in meters squared. The concentration of 25(OH)D_3_ at all stages of the experiment was measured on a Beckman Coulter DXI 800 by the chemiluminescence method (mini Vidas Blue 25 H Vitamin D total quantitative kit). Biomarkers were determined with the ELISA method on a BioTek EPOCH Instrument using Elisa Kits by SunRed for factors as NO, leptin, TMAO, sST2, VEGF-2.

### Statistical analysis

Data analysis were performed with Statistica 13.3. All results were presented as mean values with standard error of the mean (± SEM). Statistical significance was determined with the dependent t-test. The compliance of the results distribution with the normal distribution was checked using the Shapiro–Wilk test separately for the results obtained before (Time point 1) and after the 3-months cholecalciferol supplementation (Time point 2).Values of *p* ≤ 0.05 were considered statistically significant.

## Results

**Fig. 1:**
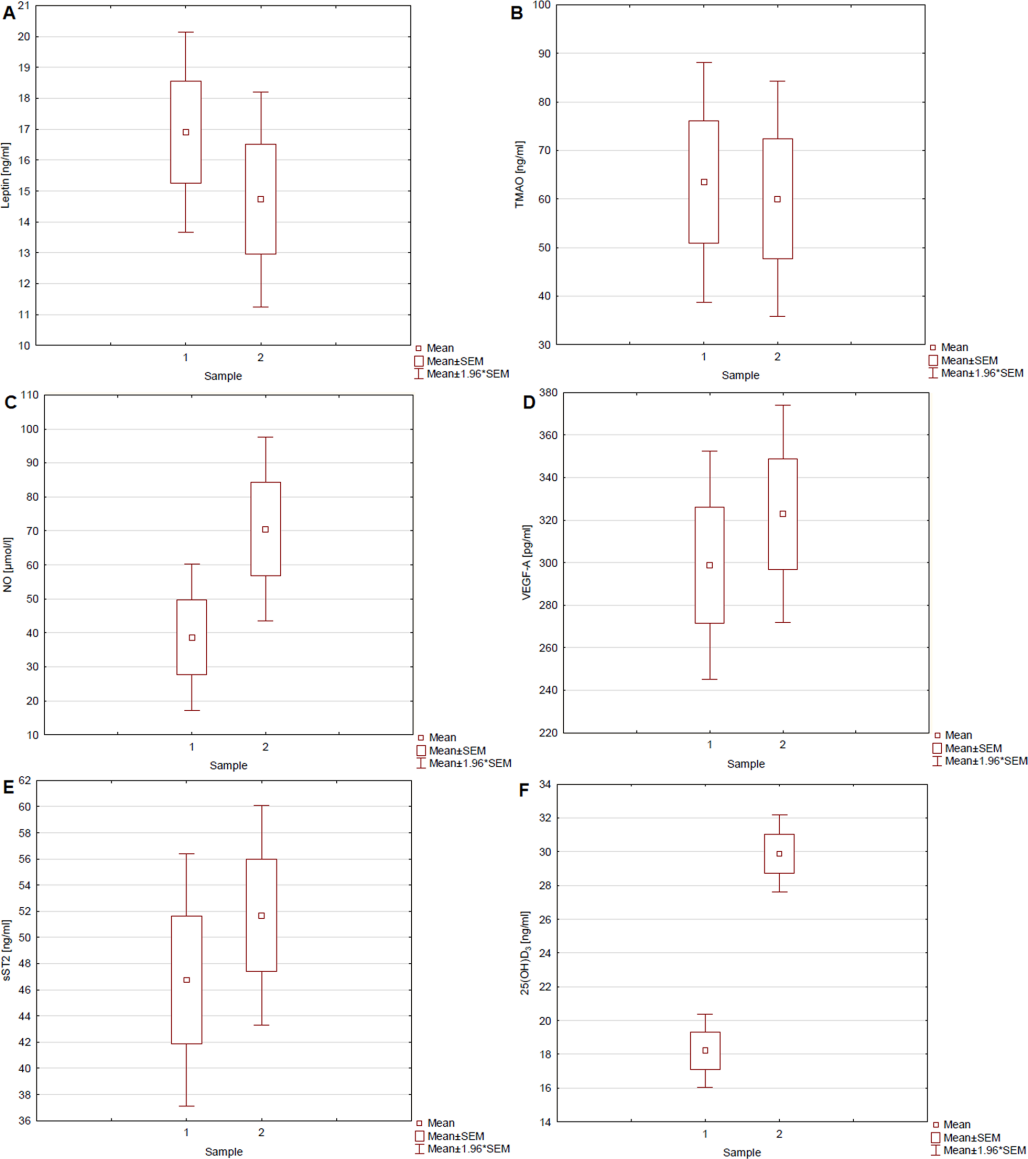
Concentration of markers and 25(OH)D_3_ before (1) and after 3 months of cholecalciferol supplementation (2). **A** leptin, *p* = 0.029; **B** TMAO, *p* = 0.022; **C** NO, *p* = 0.021; **D** VEGF-A, *p* = 0.024; **E** sST2, *p* = 0.065; **F** 25(OH)D_3_, *p* < 0.001

**Fig. 2:**
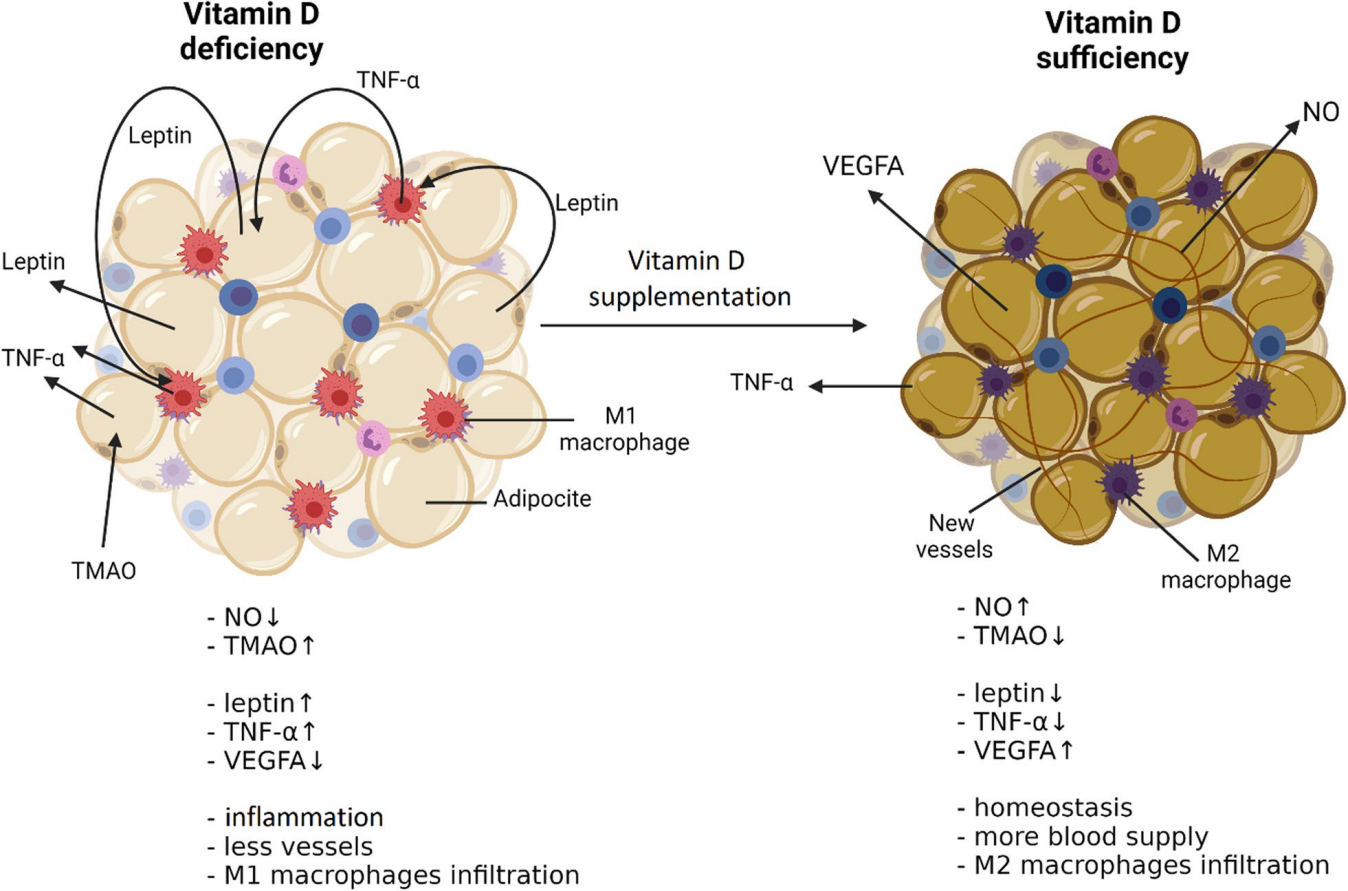
Suggested mechanism for the effect of vitamin D on adipose tissue

After 3 months of supplementation with cholecalciferol 2000 IU/day in obese people, a decrease in the level of leptin and TMAO as well as an increase in the level of NO and VEGF-A was observed. There were no statistically significant changes in serum sST2 concentration. The box plots (Fig. 1) show the concentrations of individual markers and 25(OH)D_3_ before therapy (Sample 1) and after 3 months of supplementation (Sample 2). Leptin concentration decreased from 16.90 ± 1.65 ng/ml to 14.72 ± 1.78 ng/ml and TMAO concentration from 63.41 ± 12.59 ng/ml to 59.98 ± 12.36 ng/ml. A significant increase in VEGF-A (298.81 ± 27.44 vs. 322.91 ± 26.02 pg/ml), NO (39.19 ± 10.96 vs. 70.02 ± 13.80 µmol/l) and 25(OH)D serum levels (18.22 ± 1.10 vs. 29.89 ± 1.16 ng/ml) was observed (Fig. 2).

Table 3 shows the gender distribution of 25(OH)D_3_ levels before and after supplementation. Before the supplementation, none of the patients was in the group with the optimal 25(OH)D_3_. After 3 months of treatment, the 25(OH)D_3_ level increased significantly in all the subjects, and none of the patients was in the severely deficient group. Most of the patients achieved suboptimal or optimal 25(OH)D_3_ levels (men 47.06% and 47.06%, women 50.00% and 43.75%).

**Table 3:** Distribution of 25(OH)D_3_ levels before and after supplementation according to gender (%)

	Severe deficiency		Deficiency		Suboptimal		Optimal	
	0–10 ng/ml		> 10–20 ng/ml		> 20–30 ng/ml		> 30–50 ng/ml	
	Baseline	After 3 months of supplementation	Baseline	After 3 months of supplementation	Baseline	After 3 months of supplementation	Baseline	After 3 months of supplemetation
Male	23.53	0	35.29	5.88	41.18	47.06	0	47.06
Female	6.25	0	43.75	6.25	50.00	50.00	0	43.75

According to WHO recommendations, 13 patients were classified as obesity grade I, 12 patients as obesity grade II, 7 patients as obesity grade III, and one patient as overweight (Table 4).

**Table 4:** Obesity classification

BMI (kg/m^2^)	WHO classification
< 18.5	Underweight
18.5–24.9	Normal weight
25–29.9	Overweight
30–34.9	Obesity I grade
35–39.9	Obesity II grade
> 40	Obesity III grade

## Discussion

Almost 10 years ago, Gotsman et al. published a study in which they showed that vitamin D deficiency is associated with a higher risk of death in patients with heart failure (HF) [25]. Patients who develop HF show lower serum 25(OH)D_3_ levels [25]. Researchers also observed a greater risk of subsequent HF in patients with vitamin D deficiency suffering from hypertension [26]. In the meta-analysis by Bjelakovic et al. on two-year cholecalciferol supplementation, lower mortality is observed among people with vitamin D supplementation compared to the control group [27]. A study conducted on over two million Americans indicates that a higher daily intake of cholcelciferol correlates positively with a lower risk of CVDs in men, while in women this relationship was statistically insignificant [28]. These studies may indicate an important role of vitamin D in the proper functioning of the circulatory system. Our study evaluates the effect of cholecalciferol supplementation on serum biomarkers associated with CVDs (NO, leptin, TMAO, VEGF-A, sST2) in obese patients with 25(OH)D_3_ deficiency.

We found that leptin and TMAO levels decreased after 3 months of cholecalciferol supplementation, while levels of NO and VEGF-A increased. There were no statistically significant changes in the serum concentration of sST2. It has been shown that both the observed decrease in the level of pro-inflammatory proteins and the increase in the level of VEGF-A positively correlate with a lower risk of CVD. In contrast, higher levels of sST2 indicate a higher risk of CVD. The role of NO in the peripheral regulation of the circulatory system is mainly related to the vasodilatory effect. The increase in NO synthesis leads to vasodilation, which results in an increase in blood flow and a decrease in peripheral resistance in the circulatory system. The synthesis of NO in the vessels is mainly related to the activity of endothelial nitric oxide synthase (*eNOS*), which is regulated depending on various factors, including serum 25(OH)D_3_ concentration [29, 30]. Al-Daghri et al. indicated a negative correlation between the concentration of NO and 25(OH)D_3_ in the serum of healthy adolescents [31]. On the other hand, Huang et al. proved that calcitriol improves the functioning of endothelial cells by increasing NO in patients with systemic lupus erythematosus [32]. According to the research of Andrukhova et al., vitamin D improves the functioning of the endothelium by increasing the transcription of genes encoding *eNOS* [33]. Studies in mice have shown that animals lacking *eNOS* or the neuronal nitric oxide synthase (*nNOS)* gene increase the risk of metabolic syndrome and possible vascular consequences [34]. Therefore, it seems that NO, apart from its vasodilatory effect, may play an important role in the pathogenesis of obesity.

In our study, the concentration of NO in patients was 39.19 ± 10.96 µmol/L before cholecalciferol supplementation and increased to 70.02 ± 13.80 µmol/L after three months of supplementation (*p* = 0.021). The obtained results are consistent with the data published by Huang et al., who showed that vitamin D increases the expression of *eNOS* and increases the bioavailability of NO [32]. Wolf et al. in their study assessed the relationship between serum 25(OH)D_3_ concentration and the susceptibility of skin vessels to dilation under the influence of temperature. They observed that after four weeks of oral vitamin D supplementation in a dose of 2000 IU, a significant increase in the mean concentration of 25(OH)D_3_ in the serum of the subjects was achieved (from 17.93 ± 5.24 to 26.07 ± 3.73 ng/mL, *p* = 0.04). Cholecalciferol supplementation for 4 weeks increased NO concentration and vasodilatation [35]. On the other hand, the meta-analysis by Akbari et al. showed that cholecalciferol supplementation caused a significant decrease in high-sensitivity C-reactive protein (hs-CRP), but did not affect NO levels [36].

High level of leptin, positively correlates with risk of CV events like coronary heart disease (CHD) [37], stroke [38, 39] or coronary events [40]. Clinical trials have shown that elevated serum leptin levels are associated with the risk of hypertension [41]. This peptide has been shown to activate the renin–angiotensin–aldosterone system (RAAS), increase renal tubular sodium reabsorption and stimulate sympathetic activity. In our study, the concentration of leptin in patients was measured before and after the three-month supplementation with cholecalciferol. There was a significant decrease in leptin concentration from 16.9 ± 1.65 ng/ml to 14.72 ± 1.78 ng/ml (*p* = 0.029). Manoy et al. assessed the effect of vitamin D on the levels of inflammatory markers (hs-CRP, IL-6) and leptin in patients with osteoarthritis. In this study, patients were supplemented with ergocalciferol at a dose of 40,000 IU every week for six months. There were no significant differences in the concentration of leptin and inflammatory markers [42]. However, this study should take into account the fact that people with chronic inflammatory disease took part in it. Moreover, supplementation with ergocalciferol and cholecalciferol differ from each other. Ergocalciferol has a lower affinity for vitamin D binding protein (VDBP), so its transport to the liver may be limited compared to cholecalciferol. In addition, ergocalciferol has a shorter serum half-life [43]. High serum leptin levels are associated with pathological myocardial hypertrophy and ischemia, an increased risk of serious cardiovascular events, and a poorer prognosis in patients with heart failure [44]. In a study by Mousa et al., cholecalciferol supplementation at a dose of 4000 IU daily for 16 weeks in overweight or obese people with a baseline 25(OH)D_3_ concentration ≤ 50 nmol/L did not cause significant differences in the concentration of adiponectin and leptin in the serum (*p* > 0.05) [45]. Research on the role of leptin and its effects on the body is still ongoing.

TMAO is generated from dietary choline, betaine, and L-carnitine. Multiple studies have suggested a correlation between plasma TMAO levels and the risk of CVDs. Its levels positively correlates with ongoing atherosclerosis [46], HF [47], CHD [48] and multivessel disease [49]. High levels TMAO indicate higher risk of atherosclerosis [50], first ischaemic stroke [51], as well as other CVDs and is associated with higher mortality among heart failure patients [52]. Most of the researchers show reverse correlation between VEGF-A level and CHD [53] and worse predictions for CHD patients [54]. Bernhard et al., indicate this relation may not be linear but may be reverted U-shaped [55]. According to their paper our patients are at the highest risk both before and after supplementation. According to other researchers levels after supplementation in our group are observed in healthy controls. As most studies are in contrast to the results of Berhard et al., it rather seems that lower levels of VEGF-A correlates with higher risk of CVDs. Three months of cholecalciferol therapy did not induce any statistically significant changes in serum levels of sST2. Our research results are consistent with the results of Francic et al. They showed that oral cholecalciferol supplementation at a dose of 2800 IU/day for 8 weeks, despite a statistically significant increase in serum 25(OH)D_3_ concentration of the studied patients [25(OH)D_3_ (11.3(9.2–13.5) ng/mL; *p* < 0.001)] compared to placebo, did not change the sST2 level [56].

Sarkar et al. reports that VEGF-A expression is dependent on a biochemical pathway linked to the VDR. VDR activation by vitamin D increases VEGF-A synthesis in vascular endothelial cells [57]. Research shows that the VDR is a transcription factor for the promoter of the gene encoding VEGF-A [58]. VDR is found in many cells, including adipocytes [59] and it seems justified that VEGF-A expression is also stimulated in them by a biochemical mechanism dependent on vitamin D. Biosynthesis of TMAO seems to depend on vitamin D. Obeid et al. show that TMAO plasma levels are significantly lower after cholecalciferol supplementation [60]. Adipose tissue, and more precisely WAT, apart from energy storage, also plays an endocrine role and is the largest gland in obese people [61]. It secretes many hormones, mainly leptin and to a lesser extent tumor necrosis factor α (TNFα) [62]. High levels of secreted TNFα and other pro-inflammatory cytokines indicate the existing inflammation of WAT [63]. Endothelium may be another source of proinflammatory protein synthesis, and this process also appears to depend on leptin [64]. The present study seems to show that vitamin D interrupts this pathological positive feedback mechanism of prolonged inflammation by reducing the serum leptin concentration of patients after three months of cholecalciferol supplementation. In our opinion, this may be accomplished by the inhibitory effect of vitamin D on TNFα secretion by M1 macrophages or by inhibition of the action on lipocytes for the production of leptin. The secretion of inflammatory factors can also be stimulated by TMAO [65]. The effect of cholecalciferol supplementation on the reduction of serum TMAO levels may explain its potential anti-inflammatory effect by reducing TNFα synthesis. In addition, the beneficial effect on adipose tissue results from the increase in VEGF-A concentration in WAT. As a result of angiogenesis in the WAT, there is a better blood supply and its saturation with oxygen [66]. While high levels of VEGF-A may indicate existing inflammation, there is no reason to believe that this is the case since a decrease in pro-inflammatory factors was observed in the patients studied. Mouse models of VEGF-A overexpression in WAT showed better blood supply to this tissue, and the adipocytes themselves showed features of more metabolically active cells [67, 68]. Moreover, VEGF-A reduces the expression of leptin in adipose tissue [69], which may be another part of the mechanism of the observed decrease in leptin levels. On the other hand, in murine models in which VEGF-A levels in WAT are decreased, higher levels of TNFα and leptin are observed [70]. Mahdaviani et al. reported that the thermally and metabolically active BAT adipose tissue is characterized by a higher expression of VEGF-A compared to energy storage WAT [71]. Based on the above data, it can be concluded that the correct level of 25(OH)D_3_ in the serum is essential for the maintenance of homeostasis in adipose tissue. In our opinion cholecalciferol supplementation in obese patients has a positive effect on adipose tissue and the gut microbiome. This leads to a reduction in the levels of inflammatory factors in the serum and may be responsible for a reduction in the risk of CVDs. Based on the beneficial effects of cholecalciferol supplementation on CVS shown in many studies, we believe that vitamin D may have beneficial clinical implications also in obese patients. Our study has some limitations, but in some respects it is in line with data published by other researchers. A limitation may be the methodological differences between our research and those discussed in the discussion, as well as the relatively small of test group.

## Conclusions

The data presented in our article indicate the potential effect of vitamin D on the concentration of some biomarkers in the blood serum related to CVDs. We have shown that cholecalciferol in a dose of 2000 IU/day in obese patients modifies the function of the vascular endothelium and selected parameters of inflammation. Our study provides important and valuable information at the molecular level. The three month vitamin D supplementation was associated with a decrease in TMAO and leptin levels. Supplementation was associated with an increase in NO and VEGF-A. There was no statistically significant change in sST2 concentration. The results of our study are consistent with the results of some researchers, but the data in the literature remain inconclusive. Further studies will verify whether the intervention undertaken by our team is significant in assessing the risk stratification of selected clinical aspects.

## Data Availability

The data presented in this study are available on request from the corresponding author.

## Abbreviations

BAT: Brown adipose tissue
CV: Cardiovascular
CVS: Cardiovascular system
CVDs: Cardiovascular diseases
CHD: Coronary heart disease
EDHF: Endothelium-derived hyperpolarizing factor
*eNOS*: Endothelial nitric oxide synthase
HF: Heart failure
IL-6: Interleukin 6
hs-CRP: High-sensitivity C-reactive protein
MI: Myocardial infraction
NAFLD: Non-alcoholic fatty liver disease
NF-κB: Nuclear factor kappa-light-chain-enhancer of activated B cells
*nNOS*: Neuronal nitric oxide synthase
NO: Nitric oxide
RAAS: Renin–angiotensin-aldosterone system
TMAO: Trimethylamine *N*-oxide
VDBP: Vitamin D binding protein
VEGF-A: Vascular endothelial growth factor A
VDR: Vitamin D receptor
sST2: Soluble suppression of tumorigenicity 2
TNFα: Tumor necrosis factor α
WAT: White adipose tissue
25(OH)D_3_: 25-Hydroxyvitamin D_3_
